# Clinical Characteristics and Outcomes of Invasive Aspergillosis in Patients with Hematological Malignancies and Transplantation and Cellular Therapies in the Contemporary Era

**DOI:** 10.1007/s11046-025-01046-1

**Published:** 2026-02-05

**Authors:** Christopher M. Lopez, Jose F. Suarez, Maria A. Mendoza, Anthony D. Anderson, Jill Lykon, Wenhui Li, Michele I. Morris, Yoichiro Natori, Mohammed Raja, Lazaros J. Lekakis, Amer Beitinjaneh, Antonio Jimenez, Mark Goodman, Trent P. Wang, Jay Spiegel, Noa G. Holtzman, Denise Pereira, Damian Green, Krishna V. Komanduri, Jose F. Camargo

**Affiliations:** 1 Division of Infectious Diseases, Department of Medicine, University of Miami Miller School of Medicine, Miami, FL USA; 2 Division of Infectious Diseases, Department of Medicine, University of Utah, Salt Lake City, UT USA; 3 Department of Pharmacy, Sylvester Comprehensive Cancer Center, University of Miami, Miami, FL USA; 4 Department of Pharmacy, University of Miami, Miami, FL USA; 5 Division of Transplantation & Cellular Therapy, Sylvester Comprehensive Cancer Center, University of Miami Hospital and Clinics, Miami, FL USA; 6 Division of Hematology and Oncology, Department of Medicine, University of California San Francisco, San Francisco, CA USA

**Keywords:** Invasive aspergillosis, Hematological malignancy, Hematopoietic cell transplantation, Galactomannan, Mortality

## Abstract

**Background:**

Despite important advancements in diagnostic modalities, routine use of therapeutic drug monitoring (TDM) and newer antifungal therapies, there is a paucity of contemporary data regarding clinical characteristics and outcomes of invasive aspergillosis (IA) in the United States.

**Methods:**

Single-center, retrospective cohort study of hospitalized patients between 2015 and 2020, who had active hematological malignancy (HM) or had undergone transplantation and cellular therapy (TCT) and had probable or proven IA.

**Results:**

Sixty-two patients with probable or proven IA, including 21 HM and 41 TCT, were identified. Forty-four percent of the cases corresponded to breakthrough IA. Bronchoalveolar lavage galactomannan was ≥ 1 in 71% and ≥ 0.5 in 88%, while serum galactomannan was ≥ 0.5 in only 34%. Among assessable patients (n = 59), 90-day partial or complete response to antifungal therapy occurred in 39%. All-cause mortality for the entire cohort was 22% at 30 days and 46% at 90 days. IA attributable mortality was 18% at 30 days and 38% at 90 days. Achieving therapeutic antifungal serum levels was associated with a reduction in all-cause mortality, while prior clinically significant CMV infection (aOR 9.65, 95% CI 1.34–69.6; *P* = 0.025) and relapsed/refractory hematological disease (aOR 8.5, 95% CI 2.23, 32.4; *P* = 0.002) were associated with higher IA attributable mortality.

**Conclusions:**

Despite advancements in diagnosis and treatment, IA remains associated with poor outcomes in hematological patients in the contemporary era. Newer antifungals and improved strategies for monitoring and prevention of IA in these vulnerable patient populations are urgently needed.

**Supplementary Information:**

The online version contains supplementary material available at 10.1007/s11046-025-01046-1.

## Introduction

Invasive aspergillosis (IA) occurs at a rate of 10–17% in patients with hematological malignancies (HM) and is the most common cause of invasive fungal infection (IFI) among recipients of hematopoietic cell transplantation (HCT) with 12-week mortality rates as high as 36% [1–7].

In recent years, an improved understanding of the genetic and immune host factors determining risk of IA after chemotherapy or transplantation have emerged [8]. Furthermore, advancements in diagnostic modalities such as *Aspergillus* galactomannan (GM) and *Aspergillus* PCR, routine use of therapeutic drug monitoring (TDM), and newer antifungal therapies (isavuconazole, and intravenous and extended release tablets of posaconazole) have emerged [9, 10]. However, description of the clinical characteristics and outcomes of HM patients with IA in North America is largely limited to historical cohorts studied in the first decade of the 2000s such as the Transplant-Associated Infections Surveillance Network (TRANSNET) registry [1] and the Prospective Antifungal Therapy (PATH) Alliance Registry [2]; or industry sponsored, therapy focused, non-inferiority randomized clinical trials comparing isavuconazole or posaconazole with voriconazole [10, 11]. More contemporary single center cohort studies of IA are available but have not exclusively evaluated HM patients [12–15].

To expand our current knowledge on IA in the contemporary era, we investigated the epidemiology, clinical characteristics and outcomes of IA in a cohort of 62 HM and transplantation and cellular therapy (TCT) patients diagnosed between the years 2015 and 2020 at our institution.

## Methods

### Patients and Study Design

This was a single-center retrospective study conducted at the University of Miami Hospital and the Sylvester Comprehensive Cancer Center. Study subjects were "adult patients aged 18 years or older who had HM or had undergone TCT including chimeric antigen receptor (CAR)-T cell therapy or HCT, were hospitalized between 2015 and 2020 and diagnosed with probable or proven IA. All IA cases were reviewed and adjudicated by three infectious diseases specialists (J.S., C.L., and J.F.C) before being included in the study for analysis. Cases correspond to individual patients; for subjects with more than one episode of IA, only the initial infection was included. Informed consent was obtained from all patients. The study was approved by the Institutional Review Board, consistent with principles in the Declaration of Helsinki.

### Antifungal Prophylaxis

At our center, acute leukemia patients receive mold active prophylaxis while receiving induction chemotherapy and are transitioned to fluconazole if they have achieved remission. In allogeneic HCT anti-mold prophylaxis is given to high-risk patients including haploidentical transplants, delayed engraftment (absolute neutrophil count [ANC] ≤ 500/µL for > 3 weeks), and prolonged neutropenia (ANC ≤ 500/µL for > 2 weeks) at the time of transplant; graft-versus-host disease (GVHD) requiring systemic immunosuppression and those with previous IA. Posaconazole extended-release formulation 300 mg daily is the drug of choice; however, the definitive antifungal agent depends on a number of factors including anticipated drug-drug interactions with chemotherapy, history of hepatotoxicity, medical insurance approval, and risk for QT prolongation.

### Therapeutic Drug Monitoring

Routine TDM was performed with serum voriconazole levels obtained at day 5 (target goal > 1.0 for prophylaxis, and 1.5–5.5 mg/L for treatment); serum posaconazole levels at day 7 (goal ≥ 0.7 for prophylaxis and > 1.3 mg/L for treatment); and isavuconazole levels were checked at day 7 (goal > 1 mg/L) for the patients included in this study. Antifungal serum levels were measured using liquid chromatography coupled to tandem mass spectrometry (LC/MS–MS).

### Definitions and Outcomes

Probable or proven IA was defined according to the 2008 European Organization for Research and Treatment of Cancer/Invasive Fungal Infections Cooperative Group and National Institute of Allergy and Infectious Diseases Mycosis Study group (EORTC/MSG) [16] as the study was conceived and data collection preceded the updated definitions [17]. Disseminated disease was defined as extra-pulmonary involvement of two non-contiguous organ systems (excluding sinusitis). Radiological findings were documented in accordance with the updated EORTC/MSG guidance on imaging for IA and mucormycosis [18]. Breakthrough infection was defined as IA occurring during exposure to an antifungal drug [19], regardless of documented serum levels within therapeutic range.

Primary outcomes were response to antifungal therapy, overall mortality and *Aspergillus* attributable mortality at 3 months from IA diagnosis. We used the criteria outlined by EORTC [19]/MSG [16] to define complete response at 3 months in patients with invasive mold disease including a composite of survival and resolution of all attributable symptoms and signs of disease. For partial response to therapy, we used the same criteria (i.e., survival, improvement of attributable symptoms and signs of disease, and at least 50% reduction in the diameter of the observed radiological lesion) with one modification regarding radiological response. We chose 50% (as opposed to the proposed 25%) as this value was considered more stringent, easier to assess and was used in the SECURE trial which was a non-inferiority trial of voriconazole versus isavuconazole for treatment of IA [10].

*Aspergillus* attributable mortality was defined as 1) death in the setting of worsening symptoms and signs of infection, progressive radiological findings or elevation in serum GM; or death due to an acute event involving any of the sites of infection; and 2) death was not directly related to worsening of primary disease or GVHD in those with allogeneic HCT.

### Statistics

Demographic, medical, and treatment characteristics were summarized using descriptive statistics. We used Fisher’s exact test, Mann–Whitney U test and Log-rank test when appropriate. Multivariate analysis utilizing logistic regression analysis with stepwise backward elimination was completed to evaluate risk factors associated with response to therapy and mortality. All tests were 2-sided and *P* < 0.05 was considered statistically significant. Statistical analyses were performed using either GraphPad Prism Software, Inc, version 7.03, or SPSS, version 26.

## Results

### Baseline Characteristics

A total of 62 IA patients were identified (Table S1); 21 had underlying HM and 41 received TCT, including 39 HCT (5 autologous and 34 allogeneic) and 2 CAR-T cell therapy. The median age was 57 years (range, 19–75) and 71% were males. The most common underlying diagnosis was acute myeloid leukemia (n = 24, 39%).

### Incidence and Time to Diagnosis

During the study period, a total of 1,131 patients underwent TCT at our center, including 422 allogeneic HCT, 669 autologous HCT and 40 CAR-T recipients. The incidence of IA was 8.1% among allogeneic HCT (6.9% for HLA matched related donor, 8.4% for mismatched and matched unrelated donors, and 10.3% for haploidentical donor); 5% for CAR-T, and 0.7% for autologous HCT recipients. Data on the total number of HM patients undergoing chemotherapy was not available to the authors.

In the HM group, excluding one patient who was diagnosed with IA prior to induction chemotherapy, the median time from induction to IA was 42 days (IQR, 24-81). Among those who underwent TCT, the median time from cell infusion to diagnosis of IA was 269 days (IQR, 93-422); 273 days (IQR, 116-499) for those who underwent allogeneic HCT and 141 days (IQR, 32-260) for patients who had autologous HCT or CAR-T cell therapy.

### Risk Factors for IA

In the entire cohort, 44 (71%) patients had neutropenia (ANC < 1,000 cells/μL) within 30 days of IA diagnosis; 14 (23%) had a prior documented respiratory viral infection (4 rhinovirus/enterovirus, 3 respiratory syncytial virus, 2 adenovirus, 2 influenza A, 1 human metapneumovirus, 1 coronavirus, 1 parainfluenza-3) 30 days before diagnosis of IA; and 23 (37%) had been exposed to corticosteroid doses ≥ 0.5 mg/kg of prednisone equivalent within 21 days of IA diagnosis.

Most patients with HM (n = 21) were not in remission prior to their diagnosis of IA (n = 19/21, 90%). Furthermore, 48% (10/21) of HM patients had relapsed/refractory (R/R) disease. Among TCT recipients, 10/41 (24%) had R/R disease at the time of IA diagnosis.

Among those who had TCT (n = 41), clinically significant cytomegalovirus infection (cs-CMV; defined as CMV viremia requiring therapy or tissue invasive disease) [20] within 90 days prior to IA diagnosis occurred in 12 (29%) patients. T-cell depleting agents were administered to 27/34 (79%) patients who underwent allogeneic HCT. GVHD grade 2–4 prior to IA occurred in 22/34 (65%) of allogeneic HCT patients.

### Antifungal Prophylaxis

Breakthrough IA accounted for 44% (27 out of 62) of the cases. These patients were receiving anti-mold prophylaxis at least 2 weeks prior to IA diagnosis: posaconazole (n = 13, 21%), micafungin (n = 6, 10%), voriconazole (n = 4, 6%) and isavuconazole (n = 4, 6%). TDM was performed in 71% (15/21) of those receiving prophylaxis with triazole agents, of which only one patient on voriconazole (7%) had subtherapeutic levels utilizing cut-off values of < 0.7 for posaconazole, < 1.0 for voriconazole, and < 1.0 for isavuconazole.

### Clinical and Radiological Characteristics

Applying the 2008 EORTC/MSG criteria, the 62 cases of IA corresponded to 57 probable and 5 proven diagnoses. Notably, the majority also met probable or proven EORTC/MSG criteria from the guidelines updated in 2019 (56/62, 90%).

Fifty-six patients had only pulmonary involvement (90%); 2 (3%) lung and sinus, and 4 (6%) had extrapulmonary or disseminated disease. The most frequent radiological findings on computed tomography (CT) chest included: macronodules 28/61 (46%), ground glass opacities 25/61 (41%), dense consolidation 23/61 (38%), micronodules 19/61 (31%), halo sign 9/61 (15%), mass 8/61 (13%) and cavitary lesions in 8/61 (13%).

### Fungal Biomarkers and Microbiological Results

**Fig. 1:**
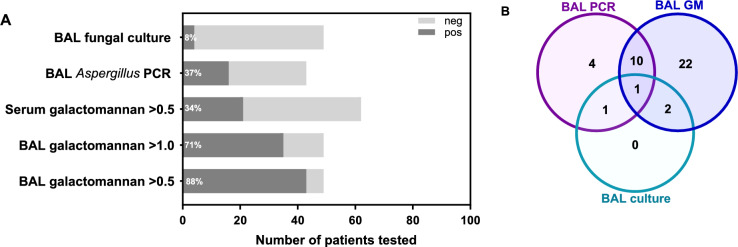
**Performance of diagnostic tests in the diagnosis of IA.** **A** Bars represent various diagnostic tests in serum and bronchoalveolar lavage (BAL) specimens for diagnosis of IA. Percentage inserts correspond to the sensitivity for a given test (i.e., proportion of IA patients with a positive test). **B** Venn diagram for patients with BAL GM> 1.0 (n = 35), positive BAL *Aspergillus* PCR (n = 16) and positive BAL fungal culture (n = 4). Note how *Aspergillus* PCR in BAL facilitated diagnosis in patients with otherwise negative BAL testing, whereas culture added no diagnostic yield to BAL galactomannan > 1.0 and BAL *Aspergillus* PCR (culture is still important for species identification and antifungal susceptibility testing)

Serum GM levels were obtained in all patients during evaluation and treatment and detected in 34% (21/62; Fig. 1) with serum GM levels ≥ 1.0 in 26% (16/62), and ≥ 0.5 but < 1.0 in 8% (5/62). Among these, 18 patients had detectable serum GM ≥ 0.5 at the time of diagnosis with a median serum GM value of 2 (IQR, 1.3–5.4). The positivity of serum GM varied according to the ANC, with a trend to lower positivity among those with ANC ≥ 1,000 versus those with ANC < 1,000 (17% vs. 41%, *P* = 0.08). Twenty of the 21 patients who had positive serum GM ≥ 0.5 received treatment; of these, 7/20 (35%) had a documented negative value in response to anti-fungal therapy, with a median time from initiation of therapy to negative serum GM of 24 days (range, 3–124). The remaining 13 patients died with increasing serum GM levels at the time of death despite antifungal therapy. Compared to those with negative serum GM, patients with positive serum GM were less likely to undergo bronchoscopy: 37/41 (90%) versus 12/21 (57%), respectively, *P* = 0.006. Among the 49 patients that underwent bronchoscopy and in whom a bronchoalveolar lavage (BAL) GM was collected, a positive result ≥ 0.5 was obtained in 43 (88%; Fig. 1); 35 (71%) had a BAL GM at 1 or above, and 8 (16%) had a BAL GM ≥ 0.5 but < 1.0.

Identification of *Aspergillus* by BAL culture was made in 4/49 (8%) patients who underwent bronchoscopy. Among those who had BAL PCR testing, identification by PCR was made in 16/43 (37%) patients (Fig. 1). Species identified by PCR included *A. fumigatus* (8/16; 50%), *A. terreus* (1/16; 6%) and other species (7/16; 44%). Including all clinical specimens at the time of diagnosis (e.g., sinus, BAL, and skin), *Aspergillus* spp. grew in culture in 11 patients, one of which was not identified at the species level. *A. fumigatus* was the most common species, accounting for 60% of the cases. Other species identified by culture included *A. terreus* (n = 1; mixed infection with *A. fumigatus*), *A. calidoustus* (n = 1), *A. hiratsukae* (n = 1), *A. ochraceopetaliformis* (n = 1) and *A. hortai* (n = 1). One patient had a second episode of IA due to *A. calidoustus* identified by tissue culture and cell-free microbial DNA nearly one year after the index infection which was culture negative. Antifungal susceptibility testing for these non-*fumigatus Aspergillus* species is presented in Table S2.

### Treatment

Three of the 62 patients died before IA diagnosis and therefore received no therapy. For the remaining 59 patients, the median time from diagnosis to initiation of antifungal therapy was 2 days (range − 7 to 61, with negative value in the range corresponding to patients on empiric antifungals) with median duration of therapy of 93 days (range 1–1,182).

Combination therapy was a common practice with up to 59% (35/59) of patients concurrently receiving ≥ 2 mold active agents during their disease course. Voriconazole was the most used antifungal (48/59, 81%), including as part of combination therapy in 24/59 (41%). Micafungin was used as combination therapy with a triazole as initial therapy in 21/59 (36%) of cases. Isavuconazole was used as the sole agent for therapy in only 2 (3%) patients. Posaconazole was not used as first line agent for treatment. Liposomal amphotericin B was used in 13 patients (22%), in all of them as a component of combination therapy.

Of the 59 patients who received treatment, 85% (50/59) underwent TDM while on therapy. Ninety-two percent (46/50) of these patients had therapeutic serum levels at least once during TDM. However, therapeutic levels were not steady, and at least one subtherapeutic level was documented in 33% (15/45) of those on voriconazole; 71% (10/14) of those on posaconazole, and 17% (2/12) of those on isavuconazole. Surgery was performed in 7% (4/59) of patients; one skin and soft tissue debridement and 3 requiring sinus debridement.

### Response to Therapy

Among assessable patients, response to antifungal therapy at 3 months, either partial or complete, occurred in 23/59 (39%) patients with complete response in 6/59 (10%) patients, and partial response documented in 17/59 (29%) patients. Complete or partial response was observed in 57%, 55% and 25% of those with autologous HCT/CAR-T cell therapy, HM and allogeneic HCT respectively.

There was no statistically significant difference among responders versus non-responders with regards to age, underlying disease, type of donor, corticosteroid use, receipt of ≥ 2 immunosuppressants, cs-CMV, subtherapeutic antifungal levels during therapy, positive serum GM or extrapulmonary disease (Table 1). Breakthrough infections were more common among non-responders (53% vs. 22%; *P* = 0.03). Among HCT patients, the time to engraftment was significantly longer among non-responders (10 vs. 14 days, *P* = 0.008). There was a trend towards higher frequency of R/R disease among non-responders (42 vs. 17%; *P* = 0.09).

**Table 1:** Patient characteristics by 90-day response and mortality among treated patients (n=59)

	Non responders (n=36)	Responders (n=23)	*P* value	Non survivors (n=27)	Survivors (n=32)	*P* value
Age, median (range)	57 (19-75)	57 (26-70)	0.91	57 (30-70)	57 (19-75)	0.68
Male gender	28 (78)	14 (61)	0.24	18 (86)	20 (63)	0.12
Comorbidities^a^	8 (22)	2 (9)	0.29	3 (14)	5 (16)	>0.99
Follow up, days, median (range)	36.5 (14-572)	429 (105-1240)	**<0.0001**	31 (3-87)	313 (92-1240)	**<0.0001**
Underlying diagnosis						
*AML*	16 (44)	8 (35)	0.59	9 (43)	12 (38)	0.78
*ALL*	5 (14)	2 (9)	0.69	2 (9)	4 (13)	>0.99
*MDS*	5 (14)	3 (13)	>0.99	5 (24)	3 (9)	0.24
*DLBCL*	3 (8)	2 (9)	>0.99	1 (5)	3 (9)	>0.99
*Myelofibrosis*	2 (6)	1 (4)	>0.99	1 (5)	1 (3)	>0.99
*Other*^b^	5 (14)	7 (30)	0.19	3 (14)	9 (28)	0.32
R/R disease	15 (42)	4 (17)	0.09	14 (52)	5 (16)	**0.005**
TCT recipient	27 (75)	12 (52)	0.09	16 (59)	19 (59)	>0.99
*Allogeneic*	24 (89)	8 (67)	0.17	14 (88)	14 (74)	0.42
*Autologous*	3 (11)	2 (17)	0.63	2 (12)	3 (16)	>0.99
*CAR-T*	0	2 (17)	0.09	0	2 (10)	0.49
Donor Class^c^						
*HLA-MMUD/MUD*	14 (58)	7 (88)	0.21	9 (64)	9 (64)	>0.99
*HLA-MRD*	7 (29)	1 (12)	0.64	3 (21)	5 (36)	0.67
*Haploidentical*	3 (13)	0	0.55	2 (14)	0	0.48
T cell depletion	17 (71)	7 (88)	0.65	11 (79)	9 (64)	0.68
*ATG*	10 (59)	4 (57)	>0.99	6 (55)	6 (67)	>0.99
*Cyclophosphamide*	7 (41)	3 (43)	>0.99	5 (45)	3 (33)	>0.99
Time to engraftment, days, median (range)^c^	14 (10-53)	10 (5-17)	**0.008**	15 (10-53)	11 (5-26)	**0.04**
ANC <1,000 cells/μL^d^	25 (69)	17 (74)	0.78	14 (67)	24 (75)	0.55
Corticosteroid use^e^	15 (42)	5 (22)	0.16	7 (33)	10 (31)	>0.99
2 IS agents	12 (33)	4 (17)	0.24	5 (24)	7 (22)	>0.99
3 IS agents	6 (17)	1 (4)	0.23	2 (10)	3 (9)	>0.99
Respiratory viral infection^d^	8 (22)	4 (17)	0.75	5 (24)	6 (19)	0.74
GVHD^f^	14 (58)	6 (75)	0.67	7 (50)	10 (71)	0.44
cs-CMV^c,g^	10 (37)	1 (10)	0.12	9 (53)	1 (5)	**0.002**
Anti-mold prophylaxis	19 (53)	5 (22)	**0.029**	13 (48)	7 (22)	0.053
*Posaconazole*	8	3		3	5	
*Voriconazole*	3	1		2	1	
*Isavuconazole*	3	1		3	1	
*Micafungin*	5	0		5	0	
Disseminated IA	1 (3)	3 (13)	0.29	0 (0)	4 (13)	0.14
Serum GM >0.5	12 (33)	6 (26)	0.77	7 (33)	9 (28)	0.76
Therapeutic serum levels^h^	26 (87)	20 (100)	0.14	17 (81)	29 (100)	**0.026**

ALL, Acute lymphoblastic leukemia; AML, Acute Myeloid Leukemia; ANC, absolute neutrophil count; ATG, anti-thymocyte globulin; CAR-T, chimeric antigen receptor T-cell therapy; cs-CMV, clinically significant cytomegalovirus; DLBCL, Diffuse Large B-Cell Lymphoma; GM, galactomannan; GVHD, graft-versus host disease; HLA, human leukocyte antigen; IA, invasive aspergillosis; IS, immunosuppression; MDS, Myelodysplastic syndrome; MMUD, mismatched unrelated donor; MUD, matched unrelated donor; R/R, relapsed/refractory; TCT, transplantation and cellular therapy.

*P* value for comparison between groups by using Mann-Whitney U test or Fisher’s exact test. Numbers in bold correspond to statistically significant values defined as *P*=<0.05

^a^Comorbidities included 5 patients with diabetes mellitus on treatment, 2 patients with end stage renal disease on dialysis, 2 patients with COPD and 1 patient with HIV.

^b^Other diagnoses include Peripheral T cell Lymphoma, Multiple Myeloma, HTLV T cell Lymphoma, PTLD, Hodgkin’s Lymphoma, Aplastic anemia, Angioimmunoblastic lymphoma, Hairy Cell Leukemia, and Hepatosplenic T cell lymphoma

^c^Among HCT recipients (total n=37, non-responders n=27, responders n=10, non-survivors n=20, survivors n=17)

^d^30 days prior to IA diagnosis

^e^Prednisone use of ≥0.5 mg/kg for ≥21 days prior to diagnosis of IA

^f^Among allogeneic hematopoietic cell transplant recipients

^g^90 days prior to IA diagnosis

^h^Defined as voriconazole >1.5, posaconazole >1.3, and isavuconazole >1, percentages defined from those who had levels obtained and denominator is not reflected by total in heading.

### Mortality

**Fig. 2:**
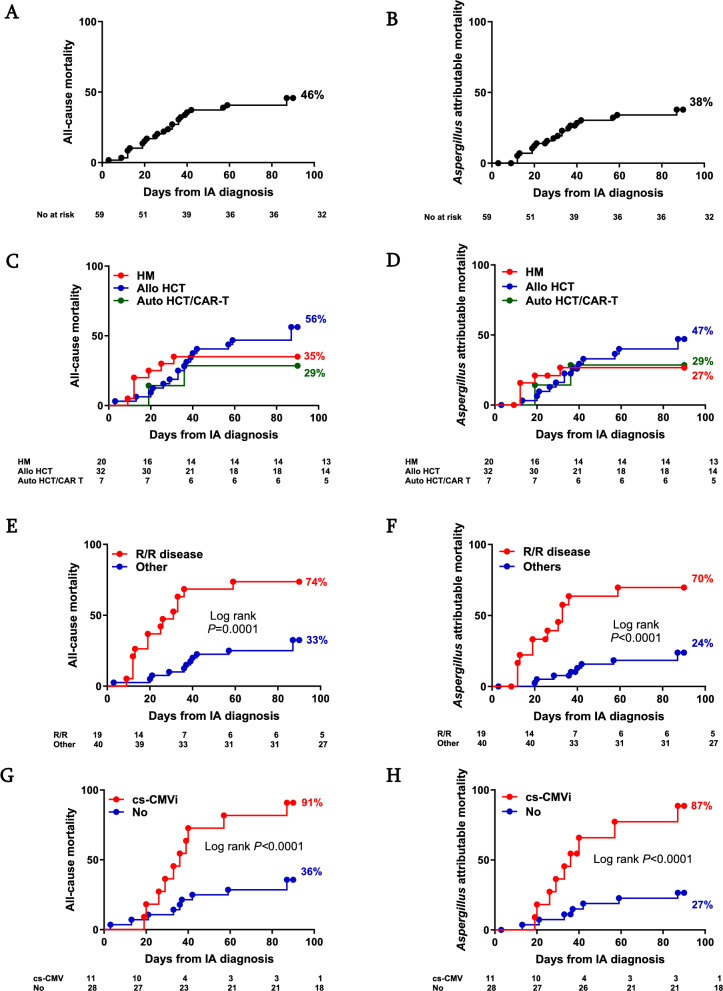
Mortality in cases of IA. Time to event curves for 3-month overall mortality (**A**, **C**, **E**, **G**) and *Aspergillus* attributable mortality (**B**, **D**, **F**, **H**) from the time of diagnosis in the entire cohort (**A**, **B**), by transplant status (**C**, **D**), by R/R status (**E**, **F**), and clinically significant CMV infection (**G**, **H**) status are shown. The number of patients at risk is shown at the bottom of each panel. Auto, autologous; Allo, allogeneic; CAR-T, chimeric antigen receptor T cell therapy; cs-CMVi, clinically significant CMV infection; HCT, hematopoietic cell transplant; IA, invasive aspergillosis; No, number; R/R, relapsed/refractory

In the entire cohort, 30 (48%) of the 62 study subjects died during the study follow up. Among those patients who received therapy (n = 59), 27 (46%) died. The median time from IA diagnosis to death was 31 days (range 3–89). All-cause mortality for the entire cohort was 22% at one month and 46% at 3 months (Fig. 2A). IA attributable mortality was 18% at one month and 38% at 3 months (Fig. 2B).

Compared to survivors, the non-survivor group included more patients with R/R malignancy (52 vs. 16%; *P* = 0.005) and had a lower proportion of patients with therapeutic serum antifungal levels while on therapy (81 vs. 100%; *P* = 0.03). Amongst HCT patients, delayed time to engraftment (15 vs. 11 days; *P* = 0.04) and cs-CMV prior to IA (53 vs. 5%; *P* = 0.002) were more common in non-survivors. There was also a trend towards higher proportion of breakthrough infections among non-survivors (48 vs. 22%; *P* = 0.053. Table 1).

Time to event analyses for 3-month mortality in HM and TCT groups are presented in Figs. 2C,D). 3-month mortality was significantly higher among those with R/R disease as well as HCT recipients with cs-CMV preceding the diagnosis of IA (Fig. 2E, G). Likewise, 3-month IA attributable mortality was significantly higher among those with R/R disease (70 vs. 24%; *P* < 0.0001, Fig. 2F) and those HCT recipients with cs-CMV within 90 days of IA diagnosis (87% vs. 27%; *P* < 0.0001 Fig. 2H).

In multivariate analyses, response to therapy was less likely to occur in patients with R/R disease (adjusted OR 0.22 95% CI 0.06–0.82; *P* = 0.024); R/R disease was also associated with increased risk of all-cause mortality (aOR 6.84, 95% CI 1.64–24.9; *P* = 0.008) and IA attributable mortality (aOR 8.5, 95% CI 2.23—32.4; *P* = 0.002). In the allogeneic HCT cohort, history of cs-CMV was associated with a tenfold increase in IA attributable mortality (aOR 9.65, 95% CI 1.34–69.6; *P* = 0.025).

## Discussion

We report the clinical characteristics and outcomes of IA in a contemporary real-world cohort of 62 patients with HM and/or TCT in a single academic institution. BAL GM was ≥ 0.5 in 88% of those who underwent bronchoscopy. This is consistent with the > 70–90% reported sensitivity of BAL GM in prior studies of HM/HCT patients [21, 22]. In contrast, serum GM was positive only in 16% of those with a positive BAL GM, and in a third of the patients in the entire cohort. Other studies have reported sensitivities <50% [23]. This highlights the importance of pursuing bronchoscopy in patients with suspected IA. Despite its low sensitivity, monitoring of serum GM can be useful to assess response to therapy; in the present study, all the patients with negative serum GM in response to antifungal therapy survived. Persistently elevated or rising levels of serum GM are an ominous sign in patients with IA that can indicate therapeutic failure, and dictate additional diagnostic evaluation and modifications in antifungal regimen [24, 25]. Correlation between serum GM, response to therapy and mortality has been previously reported in HM and HCT, and therefore GM serum levels should be used to monitor response to therapy whenever positive [22, 24–30]. Although patients in this cohort with a positive serum GM were less likely to undergo bronchoscopy (presumably as a diagnosis was already established), obtaining cultures and PCR from the infection site is highly recommended for species identification and antifungal susceptibility testing.

Consistent with the epidemiology of prior cohorts, *A. fumigatus* was the predominant species isolated [1, 3, 31–33]. However, a significant number (about 40%) of the IA cases where *Aspergillus* species could be determined were due to non-*fumigatus Aspergillus* species. Indeed, non-*fumigatus Aspergillus* species are the leading cause of breakthrough IFI among patients with HM [34], which poses a unique challenge given their tendency to multidrug resistance (Table S2) [24], 25]. The suboptimal sensitivity of fungal culture is of concern; accurate identification of *Aspergillus* at the species level along with *in vitro* susceptibility testing is critical as cryptic species present unique antifungal resistance patterns [24, 25, 35–39]. Newer molecular techniques such as cell-free microbial DNA might be useful in select cases where standard microbiological methods have been unrevealing [40–42]. This methodology was pivotal in the identification of *Aspergillus calidoustus* in one of our patients leading to timely adjustments in antifungal therapy with a good outcome [25] and recent reports support its diagnostic value [41].

Although adding serum *Aspergillus* PCR increases the sensitivity of diagnostic evaluation [40, 43–45], it is still not widely available for clinical use. In addition, one serious limitation of serum *Aspergillus* PCR is its poor sensitivity for individuals on mold-active prophylaxis [46]. Consistent with previous reports, *Aspergillus* PCR testing done in BAL was positive in 37% [47, 48]. PCR increased the diagnostic yield of BAL as there were more cases of identification of *Aspergillus* in BAL diagnosed by PCR than culture.

Amongst those with TDM data during prophylaxis, only one patient (7%) on voriconazole had subtherapeutic azole levels which suggests that current target serum drug levels might be suboptimal for prophylaxis. For example, given the observed number of breakthrough infections in patients with posaconazole levels ≥ 0.7, our institutional protocol was modified to target levels of posaconazole ≥ 1.0 mg/L for prophylaxis. Attainment of antifungal therapeutic levels, however, remains a challenge. A recent study that included over 200 HCT patients showed that nearly half failed to attain therapeutic levels of voriconazole in the first 3 weeks of therapy, and > 30% remained off target by week four [49].

More than 40% of the IA cases reported here occurred in patients receiving anti-mold prophylaxis. Other studies have reported breakthrough fungal infections in HM patients in the range of 1–10% depending on the antifungal used for prophylaxis, with the highest prophylaxis failure rates for isavuconazole and the lowest for voriconazole [50]. Our center and other groups have reported high rates of prophylaxis failures with isavuconazole [51, 52] in patients with history of HM and TCT. In the present study we observed diminished response to antifungal therapy and a trend towards higher mortality among those with breakthrough infections, consistent with prior reports [50].

The rate of partial (29%) or complete (10%) response at 90 days in our cohort is similar to that reported in industry-sponsored randomized clinical trials [10, 11]. Furthermore, *Aspergillus* attributable mortality of 38% in our cohort closely resembles these trials: SECURE trial (30–37%) and the posaconazole versus voriconazole RCT (31–34%) [10, 11].

This study also sheds light on contemporary clinical factors associated with mortality in IA. Although it is not surprising that R/R disease was correlated with lack of response to therapy and higher mortality, the association between cs-CMV and mortality is intriguing. For decades, CMV has been linked to increased risk of opportunistic infections, including IA, and increased mortality after TCT [4, 53–57]. Whether cs-CMV is merely a surrogate marker of overt immunosuppression or directly influencing mortality in IA patients require further study. Lastly, inability to achieve therapeutic anti-fungal serum levels was associated with mortality, which underscores the importance of TDM with dose adjustments and, in selected cases, even consideration of combination therapy until triazole serum levels are at the desired target.

We observed poor outcomes in this contemporary cohort despite improvements in diagnostics, antifungal therapies and TDM over the last two decades. We propose some areas which could be optimized in the diagnostic evaluation and management of IA (Table 2). In addition, current antifungals have suboptimal efficacy, and the role of combination therapy, although useful in selected scenarios [24, 29], remains controversial. Thus, there is an unmet need for approval of novel antifungal therapies [58], some of which have already shown efficacy in difficult to treat mold infections such as fosmanogepix, [25, 59], olorofim [60], rezafungin [61] and ibrexafungerp [62]. Compelling preclinical data and observational clinical studies suggest potential benefit of azole-echinocandin combination which may be considered for severe disease as salvage therapy [28, 29, 63]. We typically favor bridging with echinocandin until triazole serum levels are therapeutic in IA patients with bulky or disseminated disease. Drug levels in the higher end of the spectrum (e.g. voriconazole > 3 mg/L) are favored in severe disease [64].

**Table 2:** Considerations for optimization of diagnostic evaluation and management of invasive aspergillosis in hematological patients

1. Consider early bronchoscopy to increase diagnostic yield^a^
2. Consider early computed tomography of the chest in febrile patients with risk factors^b^
3. Incorporate non-invasive molecular methods such as *Aspergillus* PCR or microbial cell-free DNA in blood^c^
4. Close therapeutic drug monitoring in all patients receiving triazole antifungals during prophylaxis and treatment^d^

^a^Nearly 90% of the patients had BAL GM >0.5 compared to only a third with positive serum GM. The yield of BAL is highest when bronchoscopy is performed early [66].

^b^Radiologic halo sign, considered an early radiological finding in IA, was seen in less than 20% in this cohort suggesting delays in obtaining CT chest.

^c^In cases when tissue biopsy or bronchoscopy are not feasible or when results are unrevealing.

^d^Especially in those critically ill, overweight, rapid metabolizers or in cases of drug-drug interactions (e.g., co-administration of letermovir and voriconazole). If persistently subtherapeutic levels, bridging with echinocandin until therapeutic levels achieved can be considered.

Limitations of the present study include its retrospective single-center nature design with relatively small cohort size. Patient population was heterogeneous including both TCT and HM patients; however, we tried to present specific data by subgroups. Since invasive mold infections are not common in the setting of CAR-T therapy [65], there were only two CAR-T patients in this cohort (one of them who had received autologous HCT) and further studies in this area are needed. We used 2008, instead of the 2019 EORTC/MSG definitions. These criteria were primarily developed for clinical trials; in a real-life setting, a diagnosis of IA should be strongly considered in any HM/HCT patient with a positive serum GM between 0.5 and 1.0 in the presence of well-established risk factors, an abnormal CT chest and a compatible clinical syndrome, even if that GM level does not meet the proposed mycological evidence of the updated EORTC/MSG criteria. Despite these limitations, our observations provide contemporary data on the presentation and outcomes of HM/HCT patients with IA, and emphasize the unabated high mortality associated with this infection amid important advancements in diagnostics and therapeutics in the field. Newer antifungals and novel approaches to monitoring, timely diagnosis and prevention of IA in this vulnerable patient populations are urgently needed.

## Supplementary Information

Below is the link to the electronic supplementary material.

Supplementary file1 (DOCX 19 kb)

Supplementary file2 (DOCX 18 kb)

## Data Availability

No datasets were generated or analysed during the current study.

## Abbreviations

ALL: Acute lymphoblastic leukemia
AML: Acute myeloid leukemia
ANC: Absolute neutrophil count
aOR: Adjusted odds ratio
ATG: Anti-thymocyte globulin
BAL: Bronchoalveolar lavage
CAR-T: Chimeric antigen receptor T-cell therapy
CI: Confidence interval
CMV: Cytomegalovirus
CR: Clinical remission
cs-CMV: Clinically significant CMV infection
CT: Computerized tomagraphy
DLBCL: Diffuse large B-cell lymphoma
EORTC/MSG: European organization for research and treatment of cancer/invasive fungal infections cooperative group and national institute of allergy and infectious diseases mycosis study group
GM: Galactomannan
GVHD: Graft-versus-host disease
HCT: Hematopoietic cell transplant
HLA: Human leukocyte antigen
HM: Hematological malignancy
IA: Invasive aspergillosis
IFI: Invasive fungal infection
IQR: Interquartile range
IS: Immunosuppression
LC/MS–MS: Liquid chromatography coupled to tandem mass spectrometry
MDS: Myelodysplastic syndrome
MMUD: Mismatched unrelated donor
MUD: Matched unrelated donor
PATH: Prospective antifungal therapy alliance registry
PCR: Polymerase chain reaction
R/R: Relapsed/recurrent
TCT: Transplantation and cellular therapy
TDM: Therapeutic drug monitoring
