# Supplementary material for: Clinical Characteristics and Outcomes of Invasive Aspergillosis in Patients with Hematological Malignancies and Transplantation and Cellular Therapies in the Contemporary Era
Source: Mycopathologia. 2026 Feb 5;191(2):29. doi: 10.1007/s11046-025-01046-1 (PMC12876453; doi:10.1007/s11046-025-01046-1)
Supplement: Supplementary file 1 — Supplementary file1 (DOCX 19 kb) [file 11046_2025_1046_MOESM1_ESM.docx]

| Supplementary Table 1. Patient characteristics (n=62) ^a^ | |
| --- | --- |
| Male gender | 44 (71) |
| **Age, years, median (range)** | 57 (44-66) |
| **Comorbidities** | 10 (16) |
| Follow up, days, median (range) | 99 (1-1240) |
| Underlying diagnosis |  |
| *Acute myeloid leukemia* | 24 (39) |
| *Acute lymphoblastic leukemia* | 8 (13) |
| *Myelodysplastic syndrome* | 8 (13) |
| *Diffuse Large B-Cell Lymphoma* | 5 (8) |
| *Myelofibrosis* | 4 (6) |
| *Other ^b^* | 13 (21) |
| **Disease in CR at diagnosis of IA ^c^** | 23 (37) |
| Disease R/R at diagnosis of IA ^c^ | 20 (32) |
| Hematological malignancy | 21 (34) |
| Type of TCT | 41 (66) |
| *Allogeneic* | 34 (83) |
| *Autologous* | 5 (12) |
| *CAR-T* | 2 (5) |
| Donor Class ^d^ |  |
| *HLA- Mismatched or matched unrelated* | 22 (65) |
| *HLA- Matched related* | 9 (26) |
| *Haploidentical* | 3 (9) |
| T-cell depletion ^d^ | 27 (79) |
| *ATG depletion* | 15 (56) |
| *Cyclophosphamide* | 10 (44) |
| **Time to engraftment, days, median (range)** | 12 (5-53) |
| **ANC <1,000 cells/ μL ^e^** | 44 (71) |
| **Corticosteroid use ^f^** | 23 (37) |
| Two immunosuppressants at IA diagnosis | 18 (29) |
| Three immunosuppressants at IA diagnosis | 8 (13) |
| Respiratory viral infection ^e^ | 14 (23) |
| GVHD grade 2-4 ^d^ | 22 (65) |
| cs-CMV within 90 days of IA ^d^ | 12 (29) |
| **Anti-mold prophylaxis** | 27 (44) |
| *Posaconazole* | 13 (21) |
| *Voriconazole* | 4 (6) |
| *Isavuconazole* | 4 (6) |
| *Micafungin* | 6 (10) |

Abbreviations: ANC, absolute neutrophil count; ATG, anti-thymocyte globulin; CR, clinical remission; cs-CMV, clinically significant CMV; CAR-T, Chimeric Antigen Receptor Therapy; HCT, hematopoietic cell transplantation; HLA, human leukocyte antigen; IA, invasive aspergillosis; GVHD, graft-versus-host disease; R/R, relapsed/refractory; TCT, transplantation and cellular therapy;

a. Data presented as number (percentage), unless stated otherwise.

b. Other diagnoses: Peripheral T cell Lymphoma, Multiple Myeloma, HTLV T cell Lymphoma, Hodgkin’s Lymphoma, Aplastic anemia, Angioimmunoblastic lymphoma, Hairy Cell Leukemia, Hepatosplenic T cell lymphoma

c. Complete remission of primary disease at time of IA diagnosis in both HM and HCT populations

d. Among allogeneic HCT recipients

e. Within 30 days prior to diagnosis of IA

f. Prednisone use of ≥0.5 mg/kg for ≥21 days prior to diagnosis of IA
