# Supplementary material for: Clinical Characteristics and Outcomes of Invasive Aspergillosis in Patients with Hematological Malignancies and Transplantation and Cellular Therapies in the Contemporary Era
Source: Mycopathologia. 2026 Feb 5;191(2):29. doi: 10.1007/s11046-025-01046-1 (PMC12876453; doi:10.1007/s11046-025-01046-1)
Supplement: Supplementary file 2 — Supplementary file2 (DOCX 18 kb) [file 11046_2025_1046_MOESM2_ESM.docx]

**Supplementary Table 2** . Antifungal susceptibility testing (µg/mL) for clinical isolates of non-*fumigatus* *Aspergillus* species ^Λ^

|  | **AMB** | **MIC** | **CAS** | **VOR** | **POS** | **ISA** | **ITRA** | **TERB** | **MGX** |
| --- | --- | --- | --- | --- | --- | --- | --- | --- | --- |
| ***A. hiratsukae*** | 1 | 0.03 | ND | 0.5 | 0.25 | 0.5 | ND | ND | ND |
| ***A. ochraceopetaliformis*** | >16 | ND | ND | 0.5 | 0.25 | 2 | ND | ND | ND |
| ***A. hortai*** | 0.25 | 0.015 | 0.25 | 0.5 | 0.03 | 0.5 | 0.06 | ND | ND |
| ***A. calidoustus**** | 1 | 0.5 | ND | >4 | >4 | >4 | ND | 0.5 | <0.008 |
| ***A. calidoustus***** | 0.5 | <0.015 | ND | 4 | 4 | 1 | ND | 0.125 | ND |

^Λ^ Antifungal susceptibility testing was performed in selected isolates at the Fungus Testing Laboratory, University of Texas Health Science Center at San Antonio, TX.

AMB, Amphotericin B; MIC, micafungin; CAS, caspofungin; VOR, voriconazole; POS, posaconazole; ISA, isavuconazole; ITRA, itraconazole; TERB, terbinafine; MGX, fosmanogepix; ND, no data.

*Synergy testing previously published (PMID: 34930032).

** Synergy testing previously published (PMID: 31925638).
